# Aripiprazole once-monthly 400 mg for long-term maintenance treatment of schizophrenia: a 52-week open-label study

**DOI:** 10.1038/npjschz.2015.39

**Published:** 2015-11-04

**Authors:** Timothy Peters-Strickland, Ross A Baker, Robert D McQuade, Na Jin, Anna Eramo, Pamela Perry, Brian R Johnson, Anna Duca, Raymond Sanchez

**Affiliations:** 1 Global Clinical Development (CNS), Otsuka Pharmaceutical Development & Commercialization, Inc., Princeton, NJ, USA; 2 CNS Global Medical Affairs, Otsuka Pharmaceutical Development & Commercialization, Inc., Princeton, NJ, USA; 3 Global Medical, Regulatory Affairs and Alliances, Otsuka America Pharmaceutical Inc., Princeton, NJ, USA; 4 Biometrics, Otsuka Pharmaceutical Development & Commercialization, Inc., Rockville, MD, USA; 5 Medical Affairs & Phase IV Clinical Affairs, Lundbeck LLC, Deerfield, IL, USA; 6 Global Clinical Development (CNS), Otsuka Pharmaceutical Development & Commercialization, Inc., Rockville, MD, USA

## Abstract

**Background:**

Long-term maintenance treatment with an antipsychotic is often required to prevent relapse and mitigate functional deterioration in patients with schizophrenia.

**Aims:**

This study assessed the long-term safety, tolerability, and maintenance of the therapeutic effect of aripiprazole once-monthly 400 mg (AOM 400) in patients with schizophrenia.

**Methods:**

This 52-week, open-label study included patients previously enrolled in 1 of 2 AOM 400 randomized controlled trials (RCTs) and *de novo* patients. Safety endpoints included adverse events (AEs), suicidality, extrapyramidal symptoms, injection-site pain, and clinically relevant changes in clinical and laboratory values. The primary efficacy endpoint was the percentage of stable patients at baseline who remained stable at the last visit of the AOM 400 maintenance phase. All endpoints were assessed with descriptive statistics; there were no formal planned statistical analyses.

**Results:**

Of 1,247 patients screened, 1,178 enrolled in the study (194 *de novo* and 984 patients from the RCTs) and 1,081 received maintenance treatment with AOM 400. The maintenance phase completion rate was 79.4% at 52 weeks. Treatment-emergent AEs in ⩾5% of patients during open-label AOM 400 treatment were headache (7.6%), nasopharyngitis (7.0%), anxiety (6.8%), and insomnia (6.6%). There were no clinically relevant changes in safety parameters of interest. Ninety-five percent of stable patients at baseline remained stable at their last visit during the AOM 400 maintenance phase.

**Conclusions:**

The long-term safety and tolerability profile of AOM 400 was comparable to the RCTs, and the long-term therapeutic effect was maintained.

## Introduction

Schizophrenia is a chronic condition for which long-term maintenance treatment with antipsychotic medication is critical to prevent relapse and mitigate functional deterioration.^1–4^ In an effort to improve treatment adherence by eliminating the need for daily dosing, long-acting injectable (LAI) antipsychotic formulations have been developed and have demonstrated long-term improvements in relapse rates^4,5^ and may potentially minimize functional deterioration.^2,6^

Aripiprazole once-monthly 400 mg (AOM 400), an extended-release injectable suspension of aripiprazole, is the only dopamine D_2_ partial agonist^7^ available as an LAI. AOM 400 is approved for the treatment of schizophrenia. In two randomized controlled trials (RCTs), AOM 400 delayed time to impending relapse and reduced impending relapse rates versus placebo and a subtherapeutic dose of aripiprazole once-monthly, respectively.^8,9^

The current 52-week, multicenter, open-label study evaluated the long-term effectiveness of AOM 400 as maintenance treatment in patients with schizophrenia, some of whom had received AOM 400 in RCTs. The primary objective was to evaluate the long-term safety and tolerability of AOM 400, and the secondary objective was to assess long-term maintenance of the therapeutic effect.

## Materials and methods

### Study design and patients

Patients in this study (NCT00731549), conducted from February 2009 to October 2013, were *de novo* or had participated in 1 of 2 RCTs: a 52-week placebo-controlled study (NCT00705783) and a 38-week active-controlled study (NCT00706654) that assessed AOM 400 as maintenance treatment of schizophrenia.^8,9^ The 52-week study had four treatment phases, which included an oral conversion phase (4–6 weeks; patients were cross-titrated to oral aripiprazole monotherapy if they were currently treated with another antipsychotic), oral stabilization phase (4–12 weeks; patients were stabilized on oral aripiprazole 10–30 mg/day based on predefined stability criteria), AOM 400 stabilization phase (12–36 weeks; patients were exposed to single-blind treatment with AOM 400 with concomitant oral aripiprazole for the first 2 weeks), and then the maintenance treatment phase (up to 52 weeks, patients who met predefined stability criteria were randomized 2:1 to AOM 400 or placebo). The primary endpoint, time to impending relapse/exacerbation of psychotic symptoms, was significantly delayed in patients treated with AOM 400 compared with placebo.^8^ The 38-week study had three treatment phases that included an oral conversion phase (4–6 weeks; patients were cross-titrated to oral aripiprazole monotherapy if they were currently treated with another antipsychotic), oral stabilization (8–28 weeks; patients were stabilized on oral aripiprazole 10–30 mg/day based on predefined stability criteria), and the double-blind maintenance phase (up to 38 weeks; patients were randomized 2:2:1 to AOM 400, oral aripiprazole 10–30 mg/day (an active-control group), or AOM 50 mg (a subtherapeutic dose)). The primary endpoint, the Kaplan–Meier estimated impending relapse rate from randomization to week 26, was significantly lower in the AOM 400 mg group compared with the AOM 50 mg group, and non-inferiority between AOM 400 and oral aripiprazole treatment groups was established.^9^

Eligible patients aged 18 to 65 years had a current diagnosis of schizophrenia (*Diagnostic and Statistical Manual of Mental Disorders, Fourth Edition, Text Revision* *(DSM-IV-TR)*) for ⩾3 years before screening, and per the study investigator’s opinion, required chronic antipsychotic treatment and would benefit from continuing or initiating treatment with AOM 400. The assessment of potential benefit of initiating or continuing treatment with AOM 400 was made at the discretion of study investigators on a case-by-case basis. For *de novo* patients and patients with >30 days’ lapse since concluding participation in the RCTs, exclusion criteria were similar to those of the previous RCTs.^8,9^ All patients provided written informed consent prior to study participation, and the protocol and amendments were approved by the institutional review board or independent ethics committee for each respective trial site or country.

**Figure 1:**
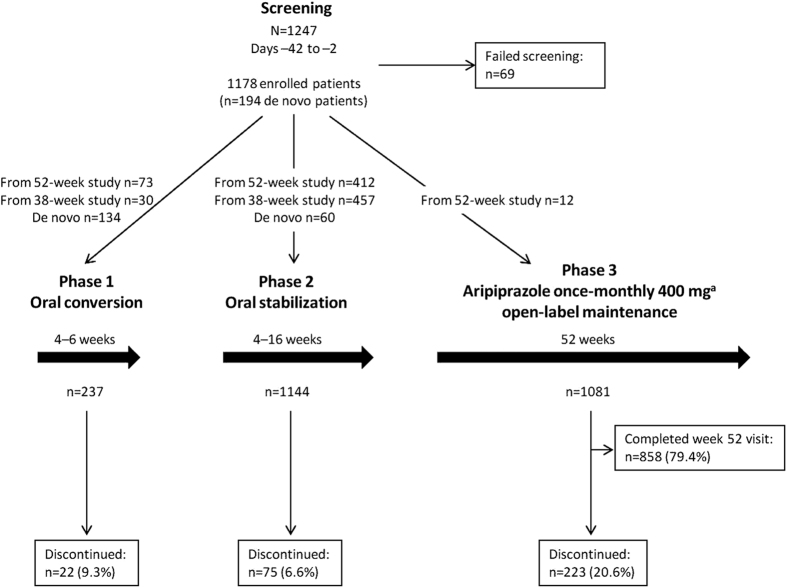
Study design and patient disposition. ^a^All patients initially received aripiprazole once-monthly 400 mg, which could be decreased to 300 mg for tolerability or returned to 400 mg as needed.

There were three consecutive treatment phases (Figure 1): an oral conversion phase (phase 1, 4–6 weeks; patients cross-titrated from their prior antipsychotic therapy to oral aripiprazole 10 or 15 mg/day and were seen weekly for assessments); an oral stabilization phase (phase 2, 4–16 weeks; patients received flexible dosing of oral aripiprazole 10–30 mg/day and were seen biweekly for assessments); and an open-label AOM 400 maintenance phase (phase 3, 52 weeks; patients were seen weekly for the first 4 weeks, biweekly for weeks 6–12, and every 4 weeks through week 52). After screening, patients who were not currently receiving oral aripiprazole (including *de novo* patients) and patients who had >30 days’ lapse since completion of or discontinuation from one of the RCTs entered the oral conversion phase and cross-titrated to oral aripiprazole. *De novo* patients currently receiving oral aripiprazole and patients from the RCTs with ⩽30 days’ lapse since concluding participation entered the current study in the oral stabilization phase and received flexible dosing of oral aripiprazole (10–30 mg).

Because the 52-week RCT was terminated after the interim analysis when the primary efficacy endpoint was met,^8^ patients who were in the conversion phase, oral stabilization phase, or single-blind AOM 400 stabilization phase of the 52-week study could enter the current study and remain in the same phase. However, all patients who were in the double-blind, placebo-controlled phase at the time of termination entered the current study in the oral stabilization phase and were restabilized on oral aripiprazole (10–30 mg), because some patients had been on placebo when the 52-week study was terminated.

All patients who met predefined stability criteria with oral aripiprazole therapy could enter the AOM 400 maintenance phase. As in the RCTs,^8,9^ stability was defined as patients meeting all of the following criteria on 1 occasion after receiving ⩾4 weeks of oral aripiprazole 10–30 mg: outpatient status; Positive and Negative Syndrome Scale (PANSS) total score ⩽80; lack of specific psychotic symptoms as measured by a score of ⩽4 on the conceptual disorganization, suspiciousness, hallucinatory behavior, and unusual thought content PANSS items; Clinical Global Impression-Severity^10^ (CGI-S) score ⩽4 (moderately ill); and a Clinical Global Impression-Severity of Suicidality^11^ (CGI-SS) score ⩽2 (mildly suicidal) on part 1 and ⩽5 (minimally worsened) on part 2.

During the maintenance phase, all patients initiated AOM 400 but could reduce their dose to 300 mg for tolerability and could return to 400 mg for efficacy, based on investigator judgment. Patients received oral aripiprazole 10–20 mg/day for the first 2 weeks after initiating AOM 400 to maintain therapeutic plasma concentrations. Thereafter, patients could receive oral aripiprazole for short-term rescue therapy and for ⩽3 episodes of impending relapse over the 52 weeks. No other antipsychotic medications were permitted during this period.

### Assessments

Safety was assessed by adverse events (AEs), including frequency, severity, seriousness, and discontinuation due to AEs. AEs ongoing at the last visit of the RCTs that increased in severity or frequency were recorded as new AEs in the current study. Potentially clinically relevant changes in vital signs, routine laboratory tests, electrocardiograms, serum prolactin concentrations, and body weight were monitored. Extrapyramidal symptoms (EPS) were assessed using the Simpson-Angus Scale^12^ (SAS), Abnormal Involuntary Movement Scale^13^ (AIMS), and Barnes Akathisia Rating Scale^14^ (BARS). Risk of suicide was assessed with the CGI-SS and the Columbia Suicide Severity Rating Scale^15^ (C-SSRS). The Columbia Classification Algorithm of Suicide Assessment^16^ was used to classify potential suicidality events that were recorded as AEs. Patients evaluated injection site pain using a visual analog scale (VAS) with ratings that ranged from 0 mm (no pain) to 100 mm (unbearable pain). Study investigators rated injection site reactions (localized pain, swelling, redness, and induration) at the most recent injection site using a 4-point categorical scale ranging from absent to severe.^17,18^

The primary efficacy endpoint was the percentage of stable patients at baseline who met all of the predefined stability criteria at the last visit of the maintenance phase. The key secondary efficacy endpoint was the proportion of patients with impending relapse^8,19^ during the maintenance phase. Impending relapse was defined as meeting ⩾1 of the following four criteria: (1) Clinical Global Impression-Improvement (CGI-I) score ⩾5 (minimally worse) AND a score >4, with an absolute increase of ⩾2, for any of the PANSS items conceptual disorganization, hallucinatory behavior, suspiciousness, or unusual thought content OR CGI-I score ⩾5 (minimally worse) AND a score >4, with an absolute increase of ⩾4, on the same combined 4 PANSS items; (2) hospitalization due to worsening of psychotic symptoms (but not for psychosocial reasons); (3) CGI-SS score of 4 (severely suicidal) or 5 (attempted suicide) on part 1 and/or 6 (much worse) on part 2; and (4) violent behavior resulting in clinically relevant self-injury, injury to another person, or property damage.

Overall effectiveness (i.e., efficacy and tolerability) was measured with the Investigator’s Assessment Questionnaire (IAQ), a scale that includes 12 items that assess efficacy and tolerability: positive symptoms, negative symptoms, other symptoms, somnolence, weight gain, signs and symptoms of prolactin elevation, akathisia, extrapyramidal symptoms, other safety or tolerability issues, cognition, energy, and mood. Each item was rated by the investigator on a 5-point scale (range: much better^1^ to much worse^5^) relative to the patient’s antipsychotic medication prior to baseline (i.e., during the oral conversion phase or at screening for *de novo* patients) and summed for a total score (range, 12–60). The IAQ was only completed by patients who underwent cross-titration during the oral conversion phase or were *de novo* patients. For the items ‘other symptoms’ and ‘other safety or tolerability issues,’ if no such symptoms or issues occurred, then a response of ‘none’ was noted. Lower scores indicate greater improvement compared with prior medication.

### Statistical analyses

The safety sample for the maintenance phase included all patients who received ⩾1 dose of AOM 400 during the maintenance phase. The efficacy sample included all patients who had ⩾1 postbaseline efficacy evaluation in the maintenance phase; baseline was defined as the last visit with available data before entering the open-label maintenance phase. Endpoints were summarized using descriptive statistics for all patients and by enrollment source. Because this was an open-label trial, no formal statistical analyses were planned. Descriptive statistics for the observed case (OC) data set were computed for selected safety endpoints in the maintenance phase: laboratory test results, vital sign parameters, electrocardiogram (ECG) parameters, SAS total score, BARS global score, AIMS movement scale score, CGI-SS severity of suicide score and change in suicidality score, and C-SSRS mean and mean change from baseline in suicidal ideation intensity score for most severe suicidal ideation.

The percentage of stable patients at baseline who remained stable at endpoint was calculated using the last visit of the maintenance phase with available data. Time to first impending relapse was plotted using a Kaplan–Meier curve. IAQ total scores and changes from baseline in PANSS total score during the maintenance phase were also assessed using descriptive statistics for the OC data set by visit for the efficacy sample.

## Results

### Patient disposition and characteristics

**Figure 2:**
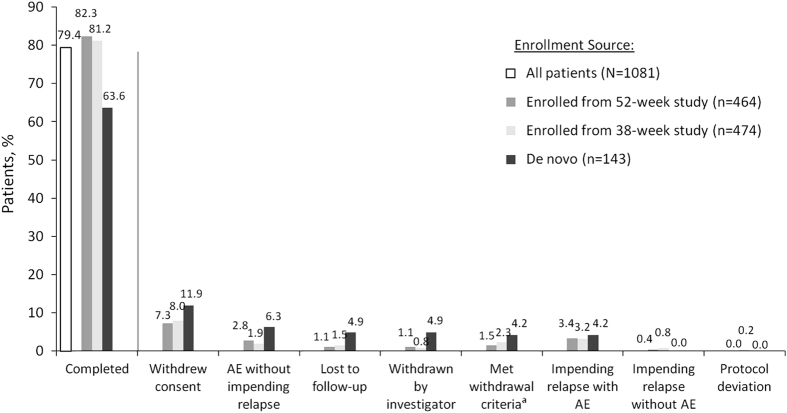
Reasons for discontinuation during the aripiprazole once-monthly 400 mg maintenance phase. ^a^Included patients who met any of the following criteria: experienced an AE, intercurrent illness, or laboratory abnormality that warranted withdrawal per investigator opinion; treated with prohibited concomitant medications; nonadherence; patient, investigator, study sponsor, or regulatory authority requested withdrawal; pregnancy; lost to follow-up; did not fulfill stability criteria on oral aripiprazole between weeks 4 and 16 of the oral stabilization phase; or met criteria for exacerbation of psychotic symptoms/impending relapse after three episodes of rescue therapy during the aripiprazole once-monthly maintenance phase. AE, adverse event.

Of 1,247 patients screened, 1,178 enrolled in the study; and 858/1,081 patients (79.4%) entered the AOM 400 maintenance phase and completed the week 52 visit (Figure 1). Patients who entered from 1 of the previous RCTs were more likely to complete the current study than *de novo* patients; 52/143 (36.4%) *de novo* patients discontinued and 171/938 (18.2%) patients who entered from the RCTs discontinued (Figure 2).

Demographic and baseline characteristics were similar across phases (Table 1). At baseline of the maintenance phase, mean PANSS total score (54.5), mean CGI-S score (3.0), and mean CGI-SS score (1.0) indicated that patients were stable and not suicidal.

### Treatment exposure and adherence

During the maintenance phase, 934/1,081 patients (86.4%) received AOM 400 mg for ⩾6 months (i.e., injections every 28 days for 7 injections) and 826/1,081 (76.4%) patients received treatment for ⩾12 months (i.e., injections every 28 days for 13 injections). In reviewing total exposure from the RCTs (i.e., including exposure during the 38-week and 52-week studies), there were 276 patients with ⩾20 months of aripiprazole once-monthly 300 or 400 mg exposure, including 36 patients with >2 years’ exposure and 1 patient who received AOM 400 for 28 months. In the current study, 92% of patients (990/1,081) initiated aripiprazole once-monthly at the 400-mg dose and had no dose change. The percentage of patients who initiated AOM 400 and had no dose change ranged from 84% for *de novo* patients to 92 and 93% for patients who entered from the 38-week and 52-week RCTs, respectively.

During the first 2 weeks of the maintenance phase, the mean (s.d.) dose of concomitant oral aripiprazole was 11.5 (2.8) mg/day. During the maintenance phase, 77/1,081 patients (7.1%) received oral aripiprazole as rescue therapy, including 17/143 (11.9%) *de novo* patients, 28/464 (6.0%) and 32/474 (6.8%) from the 52-week and 38-week RCTs. Few patients required two episodes (13/1,081, 1.2%) or 3 episodes (2/1,081, 0.2%) of rescue therapy. The mean (s.d.) dose of oral aripiprazole rescue therapy for the first episode was 10.7 (2.1) mg/day, and the mean duration was 19.2 (7.9) days. The percentage of patients who were stable at baseline and remained stable at the last visit of the maintenance treatment phase was higher for patients who did not receive oral aripiprazole as rescue therapy (967/996, 97.1%) compared with patients who were treated with oral aripiprazole rescue therapy (51/76, 67.1%).

### Safety and tolerability

#### Adverse events

During the maintenance phase, 726/1,081 patients (67.2%) had treatment-emergent AEs (TEAEs; Table 2); most were mild or moderate in severity; 95/1,081 (8.8%) patients experienced a serious TEAE. Severe TEAEs occurred in 79/1,081 patients (7.3%): 12/143 (8%) *de novo*, 30/464 (7%) from the 52-week RCT, and 37/474 (8%) from the 38-week RCT. TEAEs led to study discontinuation in 68/1,081 patients (6.3%): 15/143 (11%) *de novo*, 29/464 (6%) from the 52-week study, and 24/474 (5%) from the 38-week study.

TEAEs that occurred in ⩾1% of patients and were classified as serious, severe, or leading to study discontinuation included psychotic disorder and schizophrenia (serious: 15/1,081 (1.4%) and 21/1,081 (1.9%), respectively; severe: 13/1,081 (1%) and 14/1,081 (1%); leading to study discontinuation: 14/1,081 (1.3%) and 18/1,081 (1.7%)). Eight deaths (8/1,081 (0.7%)) occurred during the maintenance phase; most were cardiovascular related, and none were related to study medication (myocardial infarction (*n*=2); arteriosclerosis (*n*=1); cardiorespiratory arrest due to hypovolemia, dehydration, and metastatic adenocarcinoma of the lung (*n*=1); cardiac arrest (*n*=1); ruptured cerebral aneurism (*n*=1); unexplained/natural causes (*n*=1); and metastatic renal cancer (*n*=1)).

#### Safety and tolerability parameters of interest

There were no clinically relevant mean changes from baseline in serum chemistry, hematology, urinalysis, insulin, fasting insulin, vital signs (including blood pressure, heart rate, body temperature, waist circumference, or body mass index), or ECG parameters during the AOM 400 maintenance phase. There was a moderately higher incidence of potentially clinically relevant weight gain or loss (⩾7% change from baseline) at the last visit in *de novo* patients compared with patients who were enrolled from the RCTs (Table 3). Mean weight slightly increased from baseline to last visit of the maintenance phase for the overall sample (Table 3). Mean changes from baseline in serum prolactin levels in the maintenance phase were small and comparable for all patients (Table 3). At the last visit of the maintenance phase, potentially clinically relevant prolactin concentrations (above the upper limit of normal) were reported in 2.1% (23/1,070) of all patients (3/138 (2.2%) *de novo*, 10/461 (2.2%) from the 52-week RCT, and 10/471 (2.1%) from the 38-week RCT). EPS rating scale scores remained stable throughout the maintenance phase (Table 3).

Mean CGI-SS scores were 1.0 (not at all suicidal) and mean CGI change in suicidality scores were at or near 4.0 (no change) at baseline of the maintenance phase and remained stable throughout treatment for all patients and by enrollment source. No patients completed suicide during the maintenance phase. Suicidal ideation was reported by 46/1,081 patients (4.3%). Mean (s.d.) score and change from baseline in C-SSRS suicidal ideation intensity scores were 0.2 (1.5) and 0.1 (1.4), respectively, for all patients at the last visit of the maintenance phase, indicating low severity of intensity; findings were similar by enrollment source.

Most patients rated injection site pain as minimal, and most investigators rated injection site reactions as absent from the first through the last injection. Overall, patient and investigator assessments of injection site pain and reactions (redness, swelling, and induration) improved between the first and last injections; ‘absent’ ratings (i.e., absence of pain or reaction) among patients and investigators ranged from 84.9 to 98.2% after the first injection and from 87.7 to 99.1% after the last injection.

### Efficacy outcomes

**Figure 3:**
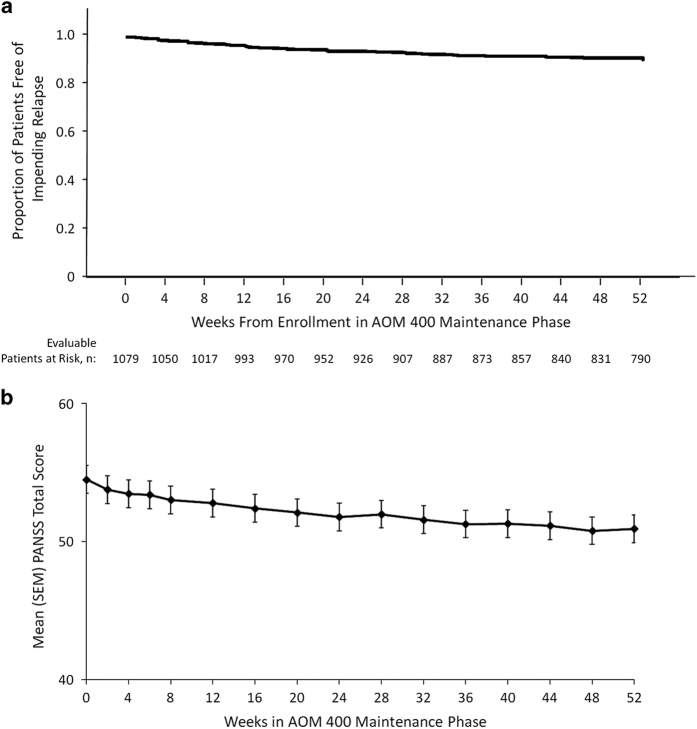
(**a**) Kaplan–Meier plot of time to first impending relapse during the aripiprazole once-monthly 400 mg maintenance phase. AOM 400, aripiprazole once-monthly 400 mg. (**b**) Mean PANSS total scores during the aripiprazole once-monthly 400 mg maintenance phase. Data are from observed cases. AOM 400, aripiprazole once-monthly 400 mg; PANSS, Positive and Negative Syndrome Scale.

Ninety-five percent (1018/1072) of patients who were stable at baseline of the maintenance phase were also stable at their last visit. The percentage of patients who were stable at baseline and remained stable was high (i.e., 98%) for nearly all study weeks thereafter, regardless of enrollment source. For most visits of the maintenance phase, the percentage of patients meeting impending relapse criteria was ⩽1%. Overall, 8.3% of patients (89/1072) met criteria for impending relapse during the maintenance phase (Figure 3a). Mean CGI-S scores remained stable from baseline to the last visit of the maintenance phase (mean (s.d.) change, −0.14 (0.70)). The mean CGI-I score at last visit, 3.35 (1.10), also indicated that, on average, patients remained stable. PANSS total scores (possible range of 30–210, with higher scores indicating more severe symptoms) also remained relatively stable during the maintenance phase (Figure 3b); mean (s.d.) change from baseline to last visit was −1.72 (10.21) and ranged from −3.40 (10.41) to −0.19 (9.19) among enrollment source groups. The mean (s.d.) IAQ total score at week 12 (first assessment) was 30.85 (5.11) and was similar at last visit (30.22 (6.00)).

## Discussion

### Main findings

Open-label maintenance treatment with AOM 400 for up to 52 weeks was safe, effective, and well tolerated in patients with schizophrenia. The completion rate was high (79.4%) and rates of metabolic abnormalities and extrapyramidal symptoms were low. The majority of patients who were stable at baseline remained stable. Most patients did not meet criteria for impending relapse, and PANSS, CGI-S, and CGI-I scores remained relatively stable, providing further evidence of the long-term maintenance of the therapeutic effect of AOM 400. Results from the current study, in which 92% of patients initiated or continued aripiprazole once-monthly therapy at 400 mg and had no dose change, confirm 400 mg^20–22^ as the effective starting and maintenance dose. Taking into account prior exposure from the RCTs, >200 patients were treated with AOM (400 mg or 300 mg) for >20 months, which provides further evidence for the well-established tolerability profile of AOM 400 in patients who remain on long-term treatment.

### Interpretation of findings in relation to previously published work

The overall AE profile was consistent with the product labeling for AOM 400 and oral aripiprazole and with the AE profile in the RCTs.^8,9^ Consistent with the known safety profile of AOM 400, none of the EPS-related TEAEs reported as akathisia were considered serious or resulted in study discontinuation, the changes in prolactin levels were not clinically relevant, a low incidence of suicidal ideation was observed, and injection site reactions were mild or absent. Likewise, the modest mean increase in weight and the low rate of potentially clinically relevant weight gain further support the long-term favorable metabolic profile.

Although the current study had no placebo group for comparison, the percentage of stable patients at baseline who met stability criteria at the last visit was numerically higher with AOM 400 (95%) in the current study compared with the placebo group in the 52-week RCT (56%) and the subtherapeutic aripiprazole once-monthly 50-mg group in the 38-week RCT (75%). In the 52-week study, 40% of patients in the placebo group met impending relapse criteria at the final analysis time point, and 22% in the aripiprazole once-monthly 50-mg group met impending relapse criteria by week 38 in the 38-week study. In the current study, 8.3% of patients treated with AOM 400 met criteria for impending relapse, which is comparable with the rates for AOM 400-treated patients during double-blind treatment in both the 52-week (10.0%) and the 38-week study (8.3%). The comparatively high rates of stability and low rates of impending relapse in our open-label study, along with the low frequency of patients who required rescue therapy during the maintenance phase, confirm that the long-term therapeutic effect of AOM 400 was maintained. The all-cause discontinuation rate with AOM 400 in the current study (21%) was similar to those in the RCTs (25% in the 52-week trial and 26% in the 38-week trial).^8,9^ In comparison with other LAI antipsychotics, the rate of all-cause discontinuation in the current study (20.6%) was lower than rates reported in a 1-year naturalistic observational study with paliperidone palmitate and a 1-year, open-label trial with risperidone LAI (35% for both),^23,24^ and similar to the discontinuation rate in a 52-week, open-label extension study with paliperidone palmitate (22%).^25^ The 20.6% all-cause discontinuation rate in the current study was also lower than rates reported in short-term, open-label studies of oral aripiprazole in the US and EU (35.0 and 25.5%, respectively).^26,27^ The higher completion rates in this long-term open-label study may reflect the stability of patients at baseline of the maintenance phase, and the study population including patients who previously tolerated AOM 400 in the prior RCTs. Although the majority of patients in our study continued into this study after participating in one of the RCTs, the all-cause discontinuation rate was also comparatively low in the *de novo* patients (36%), indicating that AOM 400 is an effective long-term treatment option. The IAQ scores at week 12 and last visit indicate that investigators rated AOM 400 as about the same or slightly better on most efficacy, safety, and tolerability items, lending further support to the long-term effectiveness of AOM 400.

### Strengths and limitations of the study

This study described the long-term clinical profile (i.e., >52 weeks in patients from RCTs) of AOM 400 as maintenance treatment in a large sample of patients with schizophrenia. The limitations of the current study, in addition to those noted in the RCTs,^8,9^ include lack of a control group, which limits the ability to attribute outcomes to treatment with AOM 400. Although self-selection of patients from the prior RCTs may limit the generalizability of our findings, we have reported all outcomes for the subpopulation of patients who entered the study *de novo*.

## Conclusions

Open-label long-term treatment with AOM 400 was well tolerated in patients with schizophrenia, and the therapeutic effect was maintained. The high completion rate and low impending relapse rate confirm that AOM 400 is an effective, safe, and well-tolerated long-term treatment option for patients with schizophrenia.

## Figures and Tables

**Table 1:** Baseline demographics and characteristics (enrolled sample)^a^^,^^b^

*Characteristic*	*Oral stabilization phase (*N*=1,144)*	*AOM 400 maintenance phase (*N*=1081)*			
		*Enrolled from 52-week study (*n*=464)*	*Enrolled from 38-week study (*n*=474)*	*De novo (*n*=143)*	*All patients (*N*=1,081)*
Men, *n* (%)	685 (59.9)	267 (57.5)	287 (60.5)	88 (61.5)	642 (59.4)
Age, y	41.0 (10.7)	40.0 (10.5)	41.7 (10.6)	43.5 (10.6)	41.2 (10.6)
					
*Race,* *n* *(%)*					
White	709 (62.0)	297 (64.0)	304 (64.1)	81 (56.6)	682 (63.1)
Black	190 (16.6)	41 (8.8)	80 (16.9)	50 (35.0)	171 (15.8)
American Indian or Alaska native	4 (0.3)	0	1 (0.2)	2 (1.4)	3 (0.3)
Asian	162 (14.2)	100 (21.6)	47 (9.9)	6 (4.2)	153 (14.2)
Native Hawaiian or other Pacific Islander	1 (0.1)	0	0	1 (0.7)	1 (0.1)
Other	78 (6.8)	26 (5.6)	42 (8.9)	3 (2.1)	71 (6.6)
					
*Ethnicity,* *n* *(%)*					
Hispanic or Latino	169 (14.8)	66 (14.2)	72 (15.2)	19 (13.3)	157 (14.5)
Not Hispanic or Latino	970 (84.8)	397 (85.6)	402 (84.8)	120 (83.9)	919 (85.0)
Unknown	5 (0.4)	1 (0.2)	0	4 (2.8)	5 (0.5)
					
Weight, kg	82.3 (21.7)	77.4 (18.9)	83.1 (21.4)	94.9 (23.6)	82.2 (21.4)
BMI, kg/m^2^	28.6 (6.9)	27.3 (6.1)	28.6 (6.7)	32.4 (7.8)	28.6 (6.8)
PANSS total score	57.9 (15.5)	55.6 (12.9)	53.4 (13.1)	54.9 (12.2)	54.5 (12.9)
CGI-S score	3.1 (1.0)	3.0 (0.9)	2.9 (0.9)	3.3 (0.8)	3.0 (0.8)
CGI-I score^c^	n/a	3.4 (0.9)	3.6 (0.7)	3.2 (0.8)	3.5 (0.8)
CGI-SS score	1.0 (0.1)	1.0 (0.1)	1.0 (0.1)	1.0 (0.1)	1.0 (0.1)

Abbreviations: AOM 400, aripiprazole once-monthly 400 mg; BMI, body mass index; CGI-I, Clinical Global Impression-Improvement; CGI-S, Clinical Global Impression-Severity; CGI-SS, Clinical Global Impression-Severity of Suicidality; n/a, not applicable; PANSS, Positive and Negative Syndrome Scale; y, years.

a Data represent the values at baseline of each respective study phase.

b Unless otherwise noted, data are presented as mean (s.d.).

c CGI-I was rated relative to end of oral stabilization.

**Table 2:** Incidence of TEAEs ⩾5% and AEs of special interest during the AOM 400 maintenance phase

*TEAE,* n *(%)*	*Enrollment source*			
	*Enrolled from 52-week study (*n*=464)*	*Enrolled from 38-week study (*n*=474)*	*De novo (*n*=143)*	*All patients (*N*=1081)*
*Any TEAE*	282 (60.8)	330 (69.6)	114 (79.7)	726 (67.2)
Headache	28 (6.0)	40 (8.4)	14 (9.8)	82 (7.6)
Nasopharyngitis	23 (5.0)	41 (8.6)	12 (8.4)	76 (7.0)
Anxiety	26 (5.6)	31 (6.5)	17 (11.9)	74 (6.8)
Insomnia	27 (5.8)	26 (5.5)	18 (12.6)	71 (6.6)
Injection site pain	13 (2.8)	31 (6.5)	8 (5.6)	52 (4.8)
Weight increased	19 (4.1)	6 (1.3)	19 (13.3)	44 (4.1)
Diarrhea	14 (3.0)	15 (3.2)	9 (6.3)	38 (3.5)
Arthralgia	13 (2.8)	12 (2.5)	9 (6.3)	34 (3.1)
Psychotic disorder	12 (2.6)	13 (2.7)	8 (5.6)	33 (3.1)
Nausea	5 (1.1)	10 (2.1)	8 (5.6)	23 (2.1)
Vomiting	6 (1.3)	7 (1.5)	8 (5.6)	21 (1.9)
				
*Any EPS-related TEAE*	35 (7.5)	43 (9.1)	19 (13.3)	97 (9.0)
Parkinsonian symptoms^a^	23 (5.0)	14 (3.0)	5 (3.5)	42 (3.9)
Akathisia	15 (3.2)	18 (3.8)	4 (2.8)	37 (3.4)
Dystonic symptoms^b^	4 (0.9)	7 (1.5)	5 (3.5)	16 (1.5)
Muscle twitching	1 (0.2)	2 (0.4)	1 (0.7)	4 (0.4)

Abbreviation: AE, adverse event; AOM 400, aripiprazole once-monthly 400 mg; EPS, extrapyramidal symptoms; TEAE, treatment-emergent AE (AEs that started after initiating study drug treatment, or an AE that was continuous from baseline and was serious, study drug related, or resulted in death, discontinuation, or interruption or reduction of study therapy).

a Included cogwheel rigidity, extrapyramidal disorders, hypertonia, hypokinesia, parkinsonism, and tremor.

b Included dystonia, muscle rigidity, muscle spasms, nuchal rigidity, oculogyric crisis, and trismus.

**Table 3:** Changes from baseline in safety endpoints of special interest during the AOM 400 maintenance phase

*Parameter*	*Enrollment source*							
	*Enrolled from 52-week study (*n*=464)*		*Enrolled from 38-week study (*n*=474)*		*De novo* (n*=143)*		*All patients (*N*=1081)*	
*Weight change at last visit*	*N*	*n* (%)	*N*	*n* (%)	*N*	*n* (%)	*N*	*n* (%)
Weight gain ⩾7%	460	59 (12.8)	473	48 (10.1)	143	27 (18.9)	1076	134 (12.5)
Weight loss ⩾7%	460	25 (5.4)	473	41 (8.7)	143	16 (11.2)	1076	82 (7.6)
								
*Weight, kg*	*N*	Mean (s.d.)	*N*	Mean (s.d.)	*N*	Mean (s.d.)	*N*	Mean (s.d.)
Baseline^a^	460	77.4 (18.9)	474	83.1 (21.4)	143	94.9 (23.6)	1077	82.2 (21.4)
Last visit Δ	460	0.8 (4.5)	473	0.3 (4.9)	143	1.0 (7.3)	1076	0.6 (5.1)
								
*Serum prolactin, ng/mL*	*N*	Mean (s.d.)	*N*	Mean (s.d.)	*N*	Mean (s.d.)	*N*	Mean (s.d.)
Baseline^a^	442	6.2 (7.0)	451	5.5 (8.1)	118	5.0 (4.4)	1011	5.8 (7.3)
Last visit Δ	439	−0.5 (5.0)	448	−0.4 (4.0)	113	−0.5 (4.1)	1000	−0.5 (4.5)
								
EPS rating scale total scores, observed cases	*N*	Mean (s.d.)	*N*	Mean (s.d.)	*N*	Mean (s.d.)	*N*	Mean (s.d.)
								
*SAS*								
Baseline	462	10.47 (1.28)	472	10.83 (1.99)	134	11.07 (2.37)	1068	10.70 (1.79)
Last visit Δ	458	−0.05 (1.19)	471	−0.07 (1.28)	132	−0.55 (2.75)	1061	−0.12 (1.51)
								
*AIMS*								
Baseline	464	0.21 (0.95)	472	0.30 (1.30)	134	0.42 (1.79)	1070	0.28 (1.24)
Last visit Δ	460	−0.05 (0.63)	471	−0.04 (0.92)	132	−0.14 (1.28)	1063	−0.05 (0.87)
								
*BARS*								
Baseline	464	0.06 (0.31)	472	0.14 (0.47)	134	0.28 (0.66)	1070	0.13 (0.45)
Last visit Δ	460	−0.02 (0.33)	471	0 (0.36)	132	−0.13 (0.71)	1063	−0.02 (0.41)

Abbreviations: Δ, change; AIMS, Abnormal Involuntary Movement Scale; AOM 400, aripiprazole once-monthly 400 mg; BARS, Barnes Akathisia Rating Scale; EPS, extrapyramidal symptoms; SAS, Simpson-Angus Scale.

*N*=Number of patients with ⩾1 baseline or postbaseline assessment of the given parameter; *n*=number of patients with weight gain or weight loss ⩾7%.

a Defined as the last evaluation before the first AOM 400 injection.
